# Differences in Expression of Genes Involved in Bone Development and Morphogenesis in the Walls of Internal Thoracic Artery and Saphenous Vein Conduits May Provide Markers Useful for Evaluation Graft Patency

**DOI:** 10.3390/ijms20194890

**Published:** 2019-10-02

**Authors:** Mariusz J. Nawrocki, Bartłomiej Perek, Patrycja Sujka-Kordowska, Aneta Konwerska, Sandra Kałużna, Piotr Zawierucha, Małgorzata Bruska, Maciej Zabel, Marek Jemielity, Michał Nowicki, Bartosz Kempisty, Agnieszka Malińska

**Affiliations:** 1 Department of Anatomy, Poznan University of Medical Sciences, 60-781 Poznań, Poland; mjnawrocki@ump.edu.pl (M.J.N.); pzawierucha@ump.edu.pl (P.Z.); mbruska@ump.edu.pl (M.B.); 2 Department of Cardiac Surgery and Transplantology, Poznan University of Medical Sciences, 61-848 Poznań, Poland; bperek@ump.edu.pl (B.P.); kardiock@ump.edu.pl (M.J.); 3 Department of Histology and Embryology, Poznan University of Medical Sciences, 60-781 Poznań, Poland; psujka@ump.edu.pl (P.S.-K.); akonwer@ump.edu.pl (A.K.); skaluzna@ump.edu.pl (S.K.); mnowicki@ump.edu.pl (M.N.); amalinsk@ump.edu.pl (A.M.); 4 Division of Anatomy and Histology, University of Zielona Góra, 65-046 Zielona Góra, Poland; maciej.zabel@gmail.com; 5 Division of Histology and Embryology, Department of Human Morphology and Embryology, Wroclaw Medical University, 50-368 Wrocław, Poland; 6 Department of Obstetrics and Gynecology, University Hospital and Masaryk University, 601 77 Brno, Czech Republic

**Keywords:** coronary artery bypass grafting, internal thoracic artery, saphenous vein, osteogenesis

## Abstract

Coronary artery bypass grafting (CABG) is one of the most efficient procedures for patients with advanced coronary artery disease. From all the blood vessels with the potential to be used in this procedure, the internal thoracic artery (ITA) and the saphenous vein (SV) are the most commonly applied as aortocoronary conduits. Nevertheless, in order to evaluate the graft patency and efficiency effectively, basic knowledge should be constantly expanding at the molecular level as well, as the understanding of predictive factors is still limited. In this study, we have employed the expressive microarray approach, validated with Real-Time Quantitative Polymerase Chain Reaction (RT-qPCR), to analyze the transcriptome of both venous and arterial grafts. Searching for potential molecular factors, we analyzed differentially expressed gene ontologies involved in bone development and morphogenesis, for the possibility of discovery of new markers for the evaluation of ITA and SV segment quality. Among three ontological groups of interest—“endochondral bone morphogenesis”, “ossification”, and “skeletal system development”—we found six genes common to all of them. *BMP6*, *SHOX2*, *COL13A1*, *CSGALNACT1*, *RUNX2,* and *STC1* showed differential expression patterns in both analyzed vessels. *STC1* and *COL13A1* were upregulated in ITA samples, whereas others were upregulated in SV. With regard to the Runx2 protein function in osteogenic phenotype regulation, the *RUNX2* gene seems to be of paramount importance in assessing the potential of ITA, SV, and other vessels used in the CABG procedure. Overall, the presented study provided valuable insight into the molecular background of conduit characterization, and thus indicated genes that may be the target of subsequent studies, also at the protein level. Moreover, it has been suggested that *RUNX2* may be recognized as a molecular marker of osteogenic changes in human blood vessels.

## 1. Introduction

Coronary artery bypass grafting (CABG), together with percutaneous coronary intervention (PCI), are both still the most efficient procedures for myocardial revascularization to treat advanced coronary artery disease (CAD). Although there are some blood vessels that can serve as grafts in reestablishing coronary circulation, the internal thoracic (mammary) artery (ITA) and the great saphenous vein (SV) are the most commonly applied in CABG patients [1,2]. In this procedure, the ITA is the conduit of choice, particularly in young individuals, while the SV is more often used in older subjects [1,3]. The ITA grafts exhibit a pivotal benefit of a favorable long-term patency rate and survival. A 10-year rate of angiographic patency reaches 90% or even more, while for vein grafts, it usually does not exceed 50% [4,5]. However, bilateral harvesting of these arteries may predispose to mediastinitis, which is a life-threatening complication [6]. The occlusion of SV grafts leads to angina recurrence, repeated acute coronary syndromes, and a need for further interventions. The subsequent surgeries are much more risky, and are associated with substantially worse early, as well as late outcomes than after primary surgery [7].

However, it should be noted that the graft patency and CABG outcomes depend on a number of factors, such as the type and quality of vessel used, the size of the recipient’s coronary arteries, or the surgeon’s skill [8]. Three main factors that can contribute to SV graft failure include acute thrombosis, neointimal hyperplasia, and eventually accelerated atherosclerosis [8]. Although the ITA has a better patency and survival, there are different comorbidities and conditions that significantly limit arterial conduit applicability [9,10,11]. Despite advances in perioperative management, better understanding of the vessel wall biology, its histological architecture, the expression of tissue markers, as well as the humoral and cellular outcomes of the process of intimal smooth muscle cell proliferation, the role of biomarkers in CABG outcome prediction is still controversial. Thus, graft patency is a result of complex and multifactoral net processes that depend on a large number of factors, some of which have not yet been well established [12]. Up to now, many predictive factors have been determined for the ITA patency rate [12,13], whereas we have still observed a limited number of such indicators for SV segments [14,15,16].

The analyses presented in this paper aim to find potential molecular factors that could be characteristic markers for both SV and ITA conduits. The performed comparison at the molecular level shows several discrepancies in the levels of gene expression belonging to different ontological groups. In this work, particular attention was paid to the ontological group, which are not the first choice at the preoperative characteristics of the vessels. We decided to describe “endochondral bone morphogenesis”, “ossification”, and “skeletal system development” gene ontology biological process (GO BP) terms also, because calcifications seen in the harvested grafts disqualify them from clinical applications. In the SV, they may result from previous inflammation or well-organized old thrombus. Macroscopic calcifications in ITA, which can be noted by a surgeon, are extremely rare. However, morphological studies of the internal thoracic artery segments harvested in patients undergoing CABG showed that even in up to 7% of them, signs of atherosclerosis may be present [17]. Nevertheless, analyzing the presented results, one can also see genes of potential significance for the examined vessels.

## 2. Results

Whole transcriptome profiling by Affymetrix microarray allowed us to analyze vascular gene expression differences. Using Affymetrix^®^ Human HgU 219 Array, we examined the expression of 49,308 transcripts. Genes with a fold change higher than abs |2| and with a corrected *p*-value lower than 0.05 were considered as differentially expressed in our study. This set of genes consisted of 1170 different transcripts.

**Figure 1:**
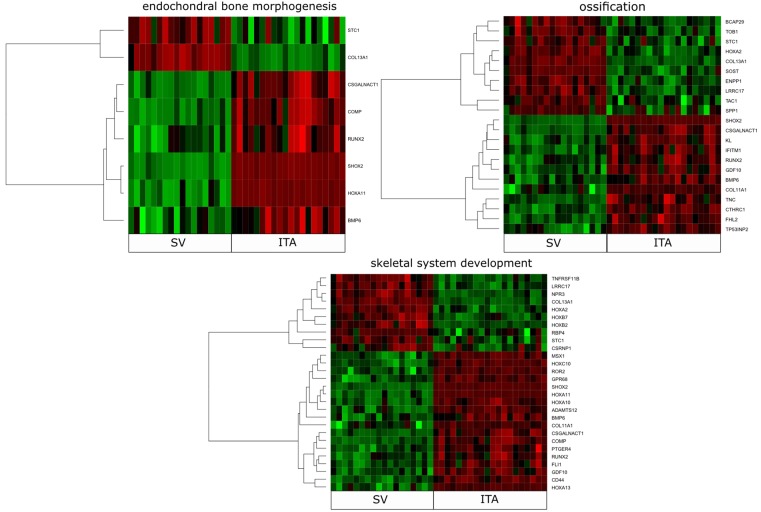
Heat map representation of differentially expressed genes belonging to “endochondral bone morphogenesis”, “ossification” and “skeletal system development” gene ontology biological process (GO BP) terms. Arbitrary signal intensity acquired from microarray analysis is represented by colors (green, higher; red, lower expression). Log2 signal intensity values for any single gene were resized to row Z-score scale (from –2, the lowest expression, to +2, the highest expression for a single gene).

DAVID (Database for Annotation, Visualization, and Integrated Discovery) software was used for the extraction of significantly enriched gene ontology (GO) terms. Upregulated and downregulated gene sets were subjected to the DAVID search separately, and only gene sets with adj. *p*-values lower than 0.05 were selected. The DAVID software analysis showed that the differently expressed genes belonged to 164 gene ontology terms. In this paper, we focused on “endochondral bone morphogenesis”, “ossification”, and “skeletal system development” gene ontology biological process terms (GO BP). A hierarchical clusterization procedure was carried out for these sets of these genes, with the results presented as heatmaps (Figure 1). There were 40 differentially expressed genes in the gene ontologies of interest. In this entire pool of genes, 24 of them showed a higher level of expression in the venous conduits, while 16 were upregulated in ITA grafts. The gene symbols, fold changes of gene expression, Entrez gene IDs, and adjusted *p*-values were shown in Table 1.

**Figure 2:**
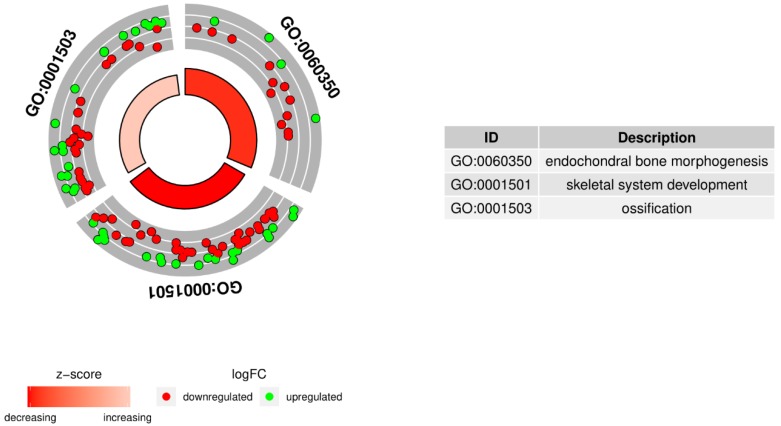
The circular visualization of the results of gene annotation enrichment analysis. The outer circle shows a scatter plot for each term of the logFC of the assigned genes. Green circles display upregulation, and red ones display downregulation. The inner circle is the representation of Z-score. The size and the color of the bar correspond to the value of the Z-score.

In order to further investigate the changes within chosen GO BP terms, we measured the enrichment levels of each selected GO BP term. The enrichment levels were expressed as Z-scores and presented as circular visualization (Figure 2).

**Figure 3:**
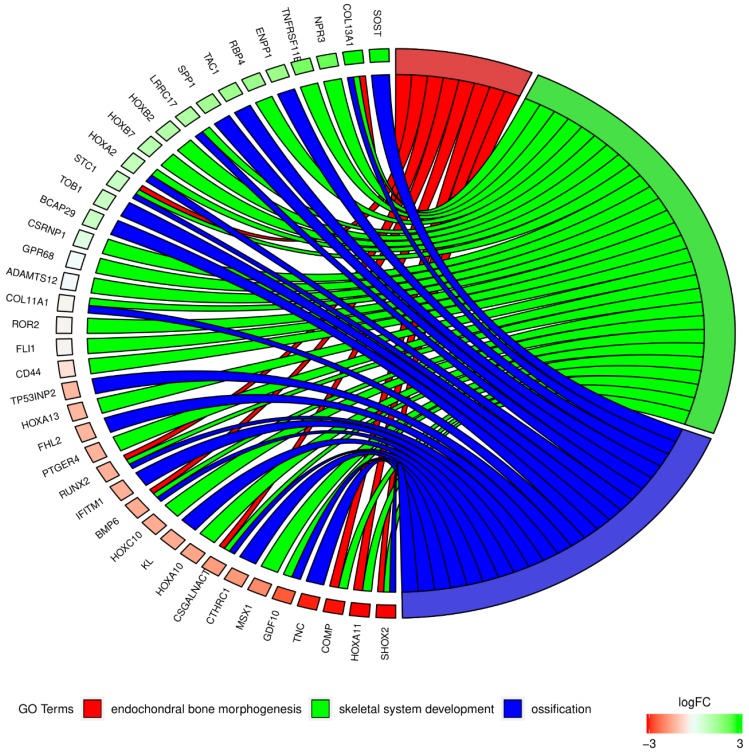
The representation of the mutual relationships between genes, which include “endochondral bone morphogenesis”, “ossification”, and “skeletal system development” GO BP terms. The ribbons show the genes belonging to the given categories. The color bars near each gene correspond to logFC between the vein and artery. The genes were sorted by logFC.

**Figure 4:**
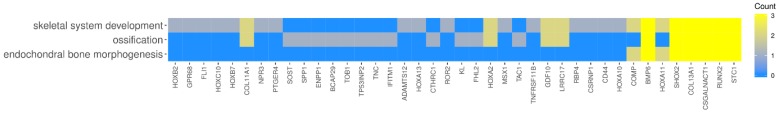
Heatmap showing the gene occurrence between genes that belong to “endochondral bone morphogenesis”, “ossification”, and “skeletal system development” GO BP terms. The yellow color indicates the gene occurrence in indicated GO BP terms. The intensity of the color correlates with the number of GO BP terms to which the selected gene belongs.

In the Gene Ontology database, genes that form one particular GO group can also belong to other different GO term categories. For this reason, we explore the gene intersections between the selected GO BP terms. The relations among those GO BP terms were presented as a circle plot (Figure 3) as well as a heatmap (Figure 4). As can be seen in the figures, six genes (*BMP6*, *SHOX2*, *COL13A1*, *CSGALNACT1*, *RUNX2,* and *STC1*) are members of all three GOs of interest. The circle plot in Figure 3 indicates genes with the highest transcript expression in the SV (such as *SHOX2* and *HOXA11*), and those that show the highest levels of mRNA in ITA grafts (*SOST* and *COL13A1*). Determining the relations between individual GO BP terms was used to select the genes used in the next stage to validate the results of microarrays.

**Figure 5:**
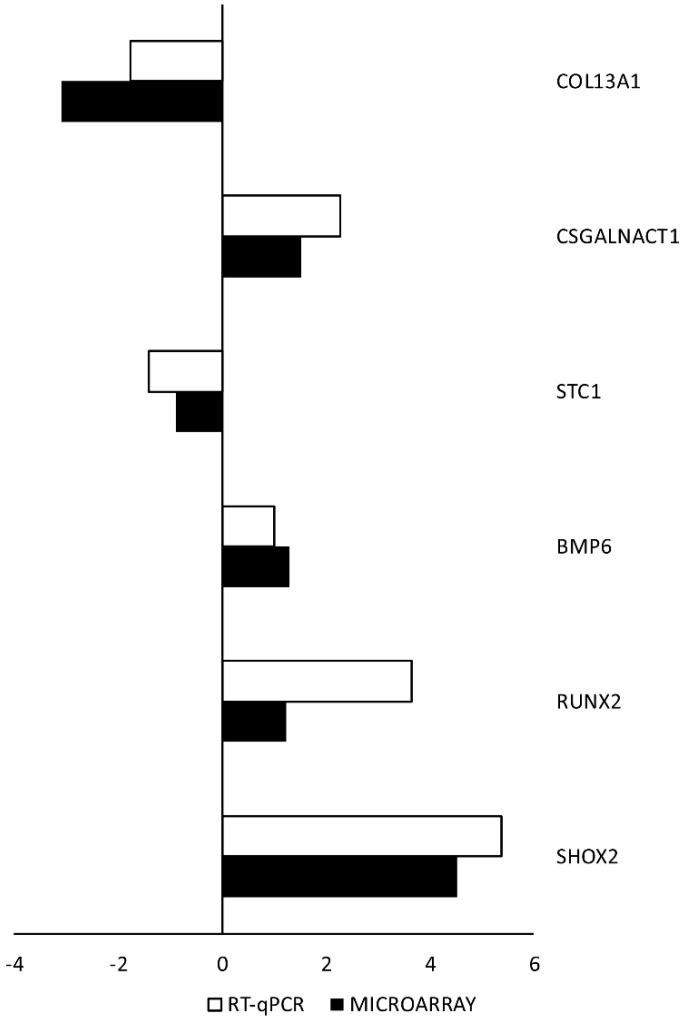
RT-qPCR quantitative validation of microarray results presented in a form of a bar graph. The graph shows the relative changes in gene expression results for veins, in relation to the transcript levels obtained from the ITA. FC was presented in its logarithmic form to provide clear comparability of the results.

RT-qPCR was performed to validate the results obtained during microarray analysis. The outcomes were presented and compared in the form of a bar graph. As can be seen, the direction of changes in expression was confirmed in all examples. However, the scale of differences in transcription levels varied between both methods analyzed (Figure 5).

**Figure 6:**
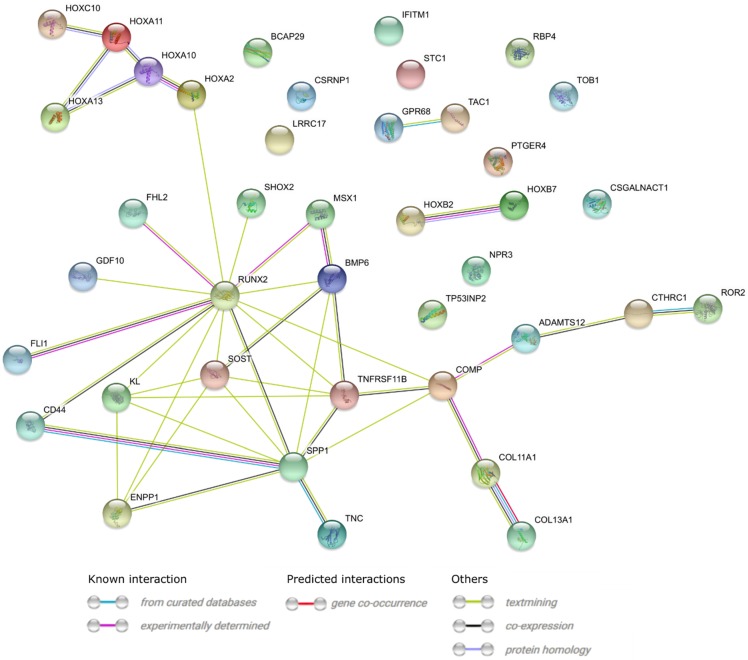
STRING-generated interaction network of genes belonging to the “endochondral bone morphogenesis”, “ossification”, and “skeletal system development” GO BP terms. The intensity of the edges reflects the strength of the interaction score.

**Figure 7:**
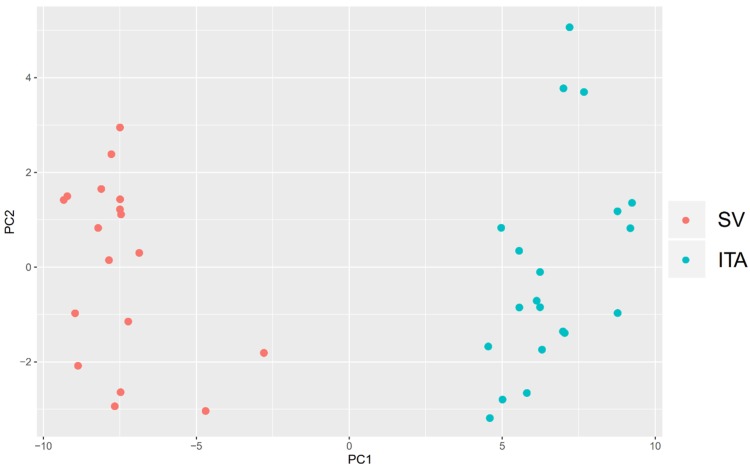
Principal component analysis (PCA) plot of significantly differentially expressed genes, based on the first two principal components’ (PC1 and PC2) loadings against each other. Each dot on the plot (red for saphenous vein (SV) and blue for internal thoracic artery (ITA)) means a single sample of the conduit used in the CABG procedure.

A STRING interaction network was generated among the chosen differentially expressed genes belonging to each of the selected GO BP terms. Using such a prediction method provided us with a molecular interaction network formed between the protein products of studied genes (Figure 6). According to the STRING database, the biggest amount of confirmed interactions characterized in the figure (also experimentally determined) can be observed with respect to the *RUNX2* gene. The result obtained further underlines the importance of *RUNX2* expression in the osteoblastic differentiation processes. Some interactions between transcription factors belonging to the family of HOX genes are also clearly noticeable. Additionally, in order to better understand the intragroup and intergroup differences in the global expression of calcification-related genes between the surgery conduits most commonly applied in CABG patients, we performed principal component analysis (PCA) of 40 genes that belongs to the selected three Gene Ontology terms (Figure 7). The dots distribution on the PCA plot clearly indicate a distinct transcriptomic profile between both CABG surgery conduits in relation to the calcification processes, which may lead to the reduced suitability of the vessel in the CABG procedure.

**Figure 8:**
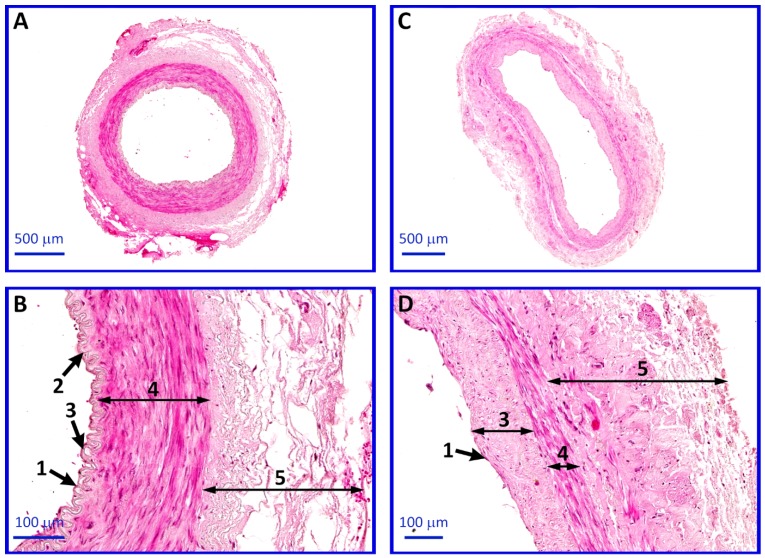
Photomicrograph representing the histological structure of internal thoracic artery (ITA; **A**,**B**) and saphenous vein (SV; **C**,**D**) segments stained with routine hematoxylin and eosin (H&E) staining. The differences in histological structure can be observed. SV is characterized by a thicker tunica adventitia and subendothelial layer compared to ITA. Arrows: 1—endothelium, 2—internal elastic lamina, 3—tunica intima, 4—tunica media, and 5—tunica adventitia.

Histological examination revealed the proper structure of ITA and SV segments. ITA is characterized by well-developed tunica media composed of smooth muscles, while in the saphenous vein, the tunica adventitia is the most prominent layer. SV is also characterized by a thickened inner layer (tunica intimia) compared to the artery (Figure 8).

## 3. Discussion

Despite the better perioperative management of CABG patients and more common application of the novel methods supporting failing organs, the early results of these procedures are still affected by adverse cardiovascular events, such as myocardial ischemia or stroke, which usually occur within the first days or weeks following operations [18,19]. The predominant reason of early acute coronary syndromes is graft failure. Thus, better understanding of the activated molecular mechanisms occurring soon after surgery may allow further a reduction of the rate of these adverse cardiovascular events. CABG, as an extensive operation, unquestionably gives rise to an important and possibly sustained activation of different pivotal molecular pathways. Researchers are increasingly devoting more attention to such changes, which are still part of a little-known area that can further increase the safety and clinical efficiency of the CABG procedure by describing the involved molecular markers. Therefore, our study aimed to investigate the transcriptomic profile of genes characterizing both ITA and SV. Employing the microarray technique, we analyzed differences at the molecular level between both blood vessels that often serve as aortocoronary grafts.

Many investigators tried to address the question concerning the possible role of single-nucleotide polymorphisms (SNPs) in graft patency and post-operative results in patients undergoing CABG. Analyzing the potential role of gene polymorphisms, numerous studies over the past few years focused on different morphological and biochemical pathways. SNPs in genes regulating oxidative stress [20], inflammatory response [21], or thrombotic pathways [22] were investigated. As an example of a changing environment after the procedure, increases in inflammatory markers can be mentioned. An increased post-operative concentration of interleukin-6 (IL-6), which has pathogenic effects on the vessel wall [23], is associated with poorer outcome [24]. However, IL6 gene variants did not affect long-term survival after CABG [21].

Among the genes belonging to GO BP terms of interest, six constitute to a specific, common element. *BMP6*, *SHOX2*, *COL13A1*, *CSGALNACT1*, *RUNX2,* and *STC1* belong to the three discussed GO BP groups: “endochondral bone morphogenesis”, “ossification”, and “skeletal system development”.

The essential function of the homeobox transcription factor *SHOX2* in the development of multiple organs, including the heart, is well established [25,26]. Its specific role is attributed to the development of the sinoatrial node (SAN), which is the primary cardiac pacemaker regulating heart-beat frequency [27]. Espinoza-Lewis et al. demonstrated that Shox2 plays an essential role in the SAN and cardiac conduction system development by controlling a genetic cascade through the repression of Nkx2-5 promoter activity [25]. Other investigators [28] also described the pivotal role of the Shox2–Nkx2-5 antagonistic mechanism operating in the cardiac venous pole, particularly in the SAN and myocardium of the pulmonary vein, in the regulation of cell fate, morphogenesis, and the distinction between pacemaker cells and working myocardium. Other research found an association of Shox2 loss-of-function mutation with enhanced susceptibility to familial atrial fibrillation (AF). Subsequent studies of AF pathogenesis similarly indicate that genetic defects may play a crucial role in the early onset of this most common sustained cardiac arrhythmia [29]. The results obtained in these studies indicate clearly higher mRNA expression in the SV compared to the ITA.

Furthermore, several other transcription factors detected in both blood vessels that can serve as grafts were described. An analysis of microarray results showed the expression of seven DNA-binding transcription factors belonging to the family of HOX genes. Interestingly, some genes exhibit higher transcript levels in SV (*HOXA11, HOXA10, HOXC10,* and *HOXA13*), whereas others were more abundantly expressed in ITA (*HOXA2, HOXB2,* and *HOXB7*). The coordinated expression of the HOX gene sub-family in both space and time is crucial for embryonic patterning. Members of this transcription factors family may regulate gene expression, morphogenesis, and differentiation [30]. Moreover, genes forming the HOX family are the main candidates for determining and maintaining region-specific physiological properties in the vascular smooth muscle cells (VSMCs) of the adult cardiovascular system. This assumption comes from the fact that clear topographic HOX expression patterns in blood vessels can be observed [31]. Furthermore, results from the Pruett laboratory indicate that proper HOX function and regulation is critical for maintaining vascular functional integrity [32].

In our study, using microarray and RT-qPCR analysis, we also found the expression of another transcription factor—the runt-related transcription factor 2 (*RUNX2*) gene, which is essential for osteoblastic differentiation, skeletal morphogenesis, and bone remodeling [33]. Runx2 evokes a process pivotal for functional disorders of the vascular system, namely VSMCs to osteogenic transdifferentiation (VOT) in a high-phosphate environment. Then, transition to an osteogenic phenotype is involved in the pathogenesis of arterial medial calcification (AMC) [34]. The induction of Runx2 and then VOT is preceded by the activation of WNT/β-catenin signaling [35,36]. Recent studies employing mice knock-out models [37] and primary rat aortic smooth muscle cells cultures [38] also confirmed the crucial role of Runx2 and downstream osteogenic pathways in VSMCs osteogenic changes, as an important factor promoting AMC. Our data indicates higher *RUNX2* transcript expression levels in SV, which may suggest greater propensity to osteogenic phenotype change in these arterial grafts compared with ITA. If this property was confirmed, it would be a factor undoubtedly decreasing the usefulness of these blood vessels as conduits in the CABG procedure.

The transcript expression member of the transforming growth factor beta (TGFB) superfamily, *BMP6*, was also detected. Bone morphogenetic protein’s (BMP) functionality is relevant to the proper form and function of blood vessels, and can function as a context-dependent pro-angiogenic cue [39,40]. According to the findings of Mouillesseaux et al. [41], new vessel formation is blocked by BMP failure via Notch regulated SMAD6 expression. Other research found BMP participation in subcortical small vessel disease (SVD) via angiogenesis promotion [42]. Similarly to the mRNA levels for the *BMP6* gene, we have also shown higher *CSGALNACT1* transcript levels in the SV. The presence of chondroitin sulfate N-acetylgalactosaminyltransferase 1 (*CSGALNACT1*) mRNA was shown in human perivascular (PV) adipocytes. Interestingly, the level of expression was higher than in subcutaneous (SQ) adipocytes [43]. Other investigators [44] described the transcript expression of this gene in aorta endothelial cells (ECs), whereas rat’s ductus arteriosus (DA) did not show the presence of this mRNA.

In combination with the above-described genes, the *STC1* gene exhibits a different pattern of expression in the compared vascular grafts. We found higher transcript levels of this gene in the arterial conduit. Stanniocalcin-1 (STC1) acts primarily as a paracrine/autocrine factor to regulate various biological functions. The STC1 hormone is normally absent in mammalian blood [45]. In a clinicopathological study of stanniocalcin-1 functions, significant elevated STC1 expression levels were described in different human cancer samples, such as tumors of lung, breast, ovary, or liver [46]. A transgenic mice model allowed observing the negative effects of STC1 and STC2 on muscle and bone development [47]. The studies of Law and Wong [48] showed the stimulatory effects of STC1 or STC2 on angiogenesis in human umbilical vein endothelial cell (HUVEC) culture. Moreover, investigators found that the action of STC1 was mediated via the VEGF/VEGFR2 pathway. Other studies indicated that STC1 plays a crucial role in the response of human brain microvascular endothelial cells to beta-amyloid peptide exposure [49].

The last of the six genes belonging to all three GO BP terms of interest was gene coding collagen type XIII alpha 1 chain, namely *COL13A1*. The function of this gene product—an alpha chain of the nonfibrillar collagens—has not been well known. It has been established that collagen type XIII is a transmembrane protein localized in cell–cell and cell–extracellular matrix (ECM) junctions [50,51]. Studies of the Miyake laboratory [52] demonstrated that the expression of COL13A1 and COL4A1 by cancer cells of human urothelial carcinoma plays a crucial role in tumor invasion through the induction of tumor budding. Other studies found that the presence of the A→C polymorphism (rs942576) of *COL13A1* was associated with the prevalence of intracerebral hemorrhage [53].

We have performed a comparative transcriptomic analysis of the two main blood vessels that serve as aortocoronary grafts in CABG patients, the internal thoracic artery and the saphenous vein, aiming to find potential molecular factors that could be helpful in assessing the suitability of individual conduits in the procedure. Analyzing the expression microarray results, we have focused on ontological groups, which were not an obvious choice when discussing blood vessel characteristics. Nevertheless, calcification may lead to the reduced suitability of the vessel in the CABG procedure. The final stage of chronic atherosclerosis is vascular calcification [54], which mimics the complex process of bone formation [55,56]. Among many others, the downregulation of calcification inhibitors, the accumulation of hydroxyapatite, the expression of osteogenic proteins (e.g., Runx2) and the transformation of vascular smooth muscle cells into osteoblast-like cells were noted [57]. Accelerated atherosclerosis is the predominant pathology responsible for aortocoronary graft failure, particularly venous. Such conclusions are also consistent with our results, because we indicate a higher level of *RUNX2* expression as a factor promoting osteogenic changes in venous grafts. In some patients, it appears in SV conduits even 6 to 12 months after surgery, but in the majority (up to 60%), it appears 10 years later [58]. Although the plaque composition in SV grafts may differ from those in the native vessels, the histopathological studies proved that calcified cartilages and amorphous calcifications were also seen in these conduits [59].

In “endochondral bone morphogenesis”, “ossification”, and “skeletal system development” GO BP terms, we have found six genes with differential expression in both vessels. Global transcriptomic analysis was performed with the use of Affymetrix microarray, and then validated with RT-qPCR. In every example, the direction of changes was confirmed, and changes in gene expression have been validated. At the same time, the scale of changes may vary significantly between the methods (such as in the case of *RUNX2*). However, this difference is not surprising, considering the different accuracy of complete transcriptomic analysis and a specific primer-based, single gene-oriented approach. It should be mentioned that the RT-qPCR is far more accurate in the quantitative analysis of transcript levels, as it targets specific gene sequences as opposed to multiple probes for different transcript variants of the same gene. Thus, whole-transcriptome screening by microarrays is mainly a qualitative, rather than quantitative study. All these genes described above play an important role in the functioning of vessels or the occurrence of undesirable states in those vessels. The transition to an osteogenic phenotype, regulated via Runx2, seems to be especially important in assessing the potential of vessel utilization in the CABG procedure. It can be assumed that, according with our transcriptome analysis, SV grafts may exhibit a higher propensity to osteogenic phenotype change compared with arterial conduits. Nevertheless, increased *RUNX2* expression levels would be a factor decreasing the usefulness of all the blood vessels used as conduits in the CABG procedure. It should be noted that although *RUNX2* expression levels analysis is valuable, calcification processes themselves play a secondary role in graft evaluation.

We hope that an analysis of the expression of genes involved in bone development and morphogenesis may help to identify patients at high risk of calcification development. It was shown in the mice model that diet was associated with a higher concentration of calcium deposits in the wall of venous grafts [59]; thus, it may be that these CABG subjects will need more aggressive medical treatment (including statins and acetylsalicylic acid) following surgery. As calcification is one of the features of occluding atherosclerotic, the future therapy oriented to downregulate the osteogenic potential of the vascular grafts may improve the long-term patency of aortocoronary conduits and simultaneously the outcomes of CABG patients.

## 4. Materials and Methods

### 4.1. Human Subjects

The procedures of the study were approved by the Local Ethical Committee of Poznań University of Medical Sciences (No. 1201/08, approved on 18/12/2008).

### 4.2. Operation Procedure and Sample Collection

In most patients, the left ITA was used to bypass the left anterior descending coronary artery (LAD). The other target coronary arteries were usually revascularized with SV grafts.

All surgeries were performed through median sternotomy. SV grafts were obtained through a full-length thigh incision over its course [60]. Pivotal points of the procedure included minimal manipulation of the graft (“no-touch” technique), avoiding extensive dilation of the conduits, using low-intensity electrocautery and the control of the branches with stainless-steel vascular clips. In all cases, the distal part of the obtained SV segment (at least 15–20 mm in length) was saved for further laboratory studies.

ITA conduits were harvested as pedicled, together with satellite veins and endothoracic fascia from the second to the sixth intercostal space. The distal end of the ITA segment was divided at the level of its bifurcation. After heparinization, ITA conduits were clipped distally, injected with 10 mL of a papaverine solution (1 mg/mL), and allowed to pharmacologically dilate. Immediately before anastomosis of the distal end of ITA to the recipient coronary artery, a 10-mm segment of the conduit was harvested for further molecular and histological tests.

The sets of the vessel samples, both SV and ITA, were immediately snap-frozen in liquid nitrogen and stored at −80 °C until RNA isolation. Another set of samples was directed for histochemical examination. Transcriptome screening analysis was performed on 18 SV and 20 ITA samples.

### 4.3. RNA Extraction and Reverse Transcription

Total RNA from the homogenized parts of the SV and ITA samples (including all the vessel layers) was isolated according to the method described by Chomczyński and Sacchi [61] and in our previous study [62,63,64]. RNA integrity was determined using denaturing agarose gel (2%) electrophoresis. Then, the RNA was quantified by measuring the optical density (OD) at 260 nm (NanoDrop spectrophotometer; Thermo Scientific, Waltham, MA, USA). RNA samples were treated with DNase I and reverse-transcribed into cDNA using an RT^2^ First Strand Kit (Qiagen, Hilden, Germany), according to the manufacturer’s protocol. Then, 500 ng of an RNA sample was used for reverse transcription.

### 4.4. Microarray Expression Study and Data Analysis

Our experiment employed 38 GeneChip^®^ HG-U219 (Affymetrix, Santa Clara, CA, USA) microarrays to simultaneously examine thousands of transcripts for each of the analyzed samples. In the first step, the total RNA (500 ng) from each pooled sample was subjected to two rounds of sense cDNA amplification (Ambion^®^ WT Expression Kit, provided by Ambion, Austin, TX, USA). The synthesis of cRNA was performed by in vitro transcription (16 h, 40 °C). Then, cRNA was purified and re-transcribed into cDNA. Subsequently, cDNA samples were used for biotin labeling and fragmentation using an Affymetrix GeneChip^®^ WT Terminal Labeling and Hybridization kit (Affymetrix). Next, the biotin-labeled samples were loaded onto and hybridized to the Affymetrix^®^ Human Genome U219 Array Strip. Hybridization was conducted at 48 °C for 20 h, employing an AccuBlock™ Digital Dry Bath (Labnet International, Inc., Edison, NJ, USA) hybridization oven. Then, microarrays were washed and stained, according to technical protocol, using an Affymetrix GeneAtlas™ Fluidics Station (Affymetrix, Santa Clara, CA, USA). The strips were scanned using an Affymetrix GeneAtlas™ Imaging Station (Affymetrix, Santa Clara, CA, USA). The scans of the microarrays were saved on hard drives as *.CEL files for downstream data analysis.

Quality control (QC) studies were performed using the Affymetrix GeneAtlas™ Instrument Control Software 2.0.0.460 (Affymetrix, Santa Clara, CA, USA), according to the manufacturer’s standards. The generated *.CEL files were subjected to further analysis performed using the R statistical language and Bioconductor package with the relevant Bioconductor libraries. To correct the background, normalize, and summarize the results, we used the robust multiarray averaging (RMA) algorithm. Assigned biological annotations were obtained from the “pd.ragene.2.1.st” library and employed for the mapping of normalized gene expression values with their symbols, gene names, and Entrez IDs, allowing to generate a complex gene data table. To determine the statistical significance of the analyzed genes, moderated t-statistics from the empirical Bayes method were performed. The obtained *p*-values were corrected for multiple comparisons using Benjamini and Hochberg’s false discovery rate and described as adjusted *p*-values. The selection of significantly altered genes was based on a *p*-value beneath 0.05 and an expression higher than two-fold. The differentially expressed gene list (separated for upregulated and downregulated genes) was uploaded to the DAVID Bioinformatics Resources 6.8 software (Database for Annotation, Visualization and Integrated Discovery) [65], where the significantly upregulated Gene Ontology (GO) terms were extracted. The selection of significantly altered GO terms was based on a *p*-value (Benajamini) < 0.05 and the volume of at least five genes.

To further investigate the chosen gene sets, we investigated their mutual relations with the GOplot package [66]. Subsequently, sets of differentially expressed genes from selected GO BP terms were applied to the STRING10 software (Search Tool for the Retrieval of Interacting Genes/Proteins) for interactions prediction. STRING is a huge database containing information on protein/gene interactions, including experimental data, computational prediction methods, and public text collections. Finally, the principal component analysis (PCA) of 40 genes that belong to the selected three Gene Ontology terms were investigated. The PCA plot of significantly differentially expressed genes has been prepared based on the first two principal components’ (PC1 and PC2) loadings against each other.

### 4.5. Real-Time Quantitative Polymerase Chain Reaction (RT-qPCR) Analysis

Determination of the transcript levels of six genes belonging to all GO BP terms of interest was conducted using a Light Cycler^®^ 96 Real-Time PCR System, Roche Diagnostics GmbH (Mannheim, Germany) with SYBR Green I as a detection dye. Each venous/arterial graft sample was tested independently. Levels of analyzed transcripts were standardized in each sample, in reference to *hypoxanthine phosphoribosyltransferase 1* (HPRT1) and *β-actin* (ACTB) as an internal control. For target cDNA quantification, we have performed relative quantification with the 2^−ΔΔ*C*^_T_ method. For each of the amplification reactions, 1 μL of cDNA solution was mixed with 9 μL of reaction master mix (5× FastStart Essential DNA Green Master (Roche Diagnostics GmbH; Mannheim, Germany) and a specific starter pair). We have used the Primer3 software for primer design (Table 2). The exon–exon design method was used as an additional method to avoid the possible amplification of genomic DNA fragments. The primers were also designed using the sequence of several transcript variants of genes of interest available in the Ensembl database. In order to confirm the specificity of the results, 2% agarose gel electrophoresis of the products was performed. An analysis of dissociation curves in equipment software provided by the manufacturer also confirmed the specificity of the amplification products obtained in the RT-qPCR analysis.

### 4.6. Histological Analysis

Immediately after harvesting, both ITA and SV segments were fixed with Bouin’s solution for 48 h. Subsequently, the collected tissues were dehydrated and embedded in paraffin blocks and then cut into 4-µm thick sections with a semi-automatic rotary microtome (Leica RM 2145, Leica Microsystems, Nussloch, Germany). Afterwards, the blood vessels sections were stained via the routine hematoxylin and eosin (H&E) method, following the protocol of: deparaffinization and rehydration with xylene, decreasing concentrations of alcohols and water, H&E staining, and dehydration with increasing concentrations of alcohols and xylene. Histological sections were evaluated under a light microscope, with selected pictures were taken using a high-resolution scanning technique and Olympus BX61VS microscope scanner (Olympus, Tokyo, Japan).

## 5. Conclusions

Our transcriptomic analysis of two conduits most commonly applied in coronary artery bypass grafting procedure, the internal thoracic artery (ITA) and the saphenous vein (SV), showed potential molecular markers of osteogenic changes in these vessels. Especially, the *RUNX2* gene seems to be of paramount importance in assessing the potential of ITA, SV and other vessels used in the CABG procedure. According to the results we obtained, venous grafts may be more exposed to osteogenic changes.

## Figures and Tables

**Table 1:** Gene symbols, fold changes in expression, Entrez gene IDs, and corrected *p*-values of studied genes.

Gene Symbol	Ratio	Adjusted *p* Value	Entrez Gene ID
*SHOX2*	−22.8240429	1.03 × 10^−27^	6474
*HOXA11*	−9.830934198	7.60 × 10^−17^	3207
*COMP*	−7.773021124	3.33 × 10^−13^	1311
*TNC*	−7.430294969	1.88 × 10^−08^	3371
*GDF10*	−5.309648509	2.16 × 10^−08^	2662
*MSX1*	−3.455950944	9.17 × 10^−13^	4487
*CTHRC1*	−3.018066571	5.20 × 10^−11^	115908
*CSGALNACT1*	−2.881617301	2.10 × 10^−12^	55790
*HOXA10*	−2.63199568	1.36 × 10^−12^	3206
*KL*	−2.458105612	2.59 × 10^−08^	9365
*HOXC10*	−2.457042843	7.09 × 10^−15^	3226
*BMP6*	−2.45216404	2.98 × 10^−07^	654
*IFITM1*	−2.349841846	2.37 × 10^−08^	8519
*RUNX2*	−2.331169268	5.34 × 10^−07^	860
*PTGER4*	−2.280369849	7.12 × 10^−09^	5734
*FHL2*	−2.216349206	6.35 × 10^−10^	2274
*HOXA13*	−2.166453813	2.16 × 10^−10^	3209
*TP53INP2*	−2.127154082	3.09 × 10^−08^	58476
*CD44*	−1.387817152	0.006540227	960
*FLI1*	–1.131519079	0.002636506	2313
*ROR2*	−1.128865754	0.004346143	4920
*COL11A1*	−1.12582207	0.000190115	1301
*ADAMTS12*	−1.054115576	0.188720272	81792
*GPR68*	−1.028071249	0.512393039	8111
*CSRNP1*	1.338139052	0.026644084	64651
*BCAP29*	1.823380549	3.52 × 10^−07^	55973
*TOB1*	1.841885719	1.99 × 10^−08^	10140
*STC1*	1.845964521	0.000109973	6781
*HOXA2*	2.01745347	5.20 × 10^−11^	3199
*HOXB7*	2.230080809	3.31 × 10^−09^	3217
*HOXB2*	2.433099517	5.86 × 10^−11^	3212
*LRRC17*	2.543411445	2.26 × 10^−10^	10234
*SPP1*	2.96640349	0.001152397	6696
*TAC1*	3.167584851	4.92 × 10^−05^	6863
*RBP4*	3.228449588	3.61 × 10^−05^	5950
*ENPP1*	3.320786599	4.83 × 10^−10^	5167
*TNFRSF11B*	4.741562441	1.54 × 10^−10^	4982
*NPR3*	5.295452922	2.59 × 10^−12^	4883
*COL13A1*	8.475105942	8.83 × 10^−18^	1305
*SOST*	12.36428646	1.01 × 10^−10^	50964

**Table 2:** Oligonucleotide sequences of primers used for RT-qPCR analysis.

Gene	Primer Sequence (5′–3′)		Product Size (bp)
*SHOX2*	F	GAAGGCCAGACCAAAATCAA	234
	R	GGCCCCTATGAGAACACCTT	
*RUNX2*	F	GGACGAGGCAAGAGTTTCAC	165
	R	GAGGCGGTCAGAGAACAAAC	
*BMP6*	F	AAGAAGGCTGGCTGGAATTT	170
	R	GAAGGGCTGCTTGTCGTAAG	
*STC1*	F	TGATCAGTGCTTCTGCAACC	242
	R	GACGAATGCTTTTCCCTGAG	
*CSGALNACT1*	F	CAGAAAGGGACAAAGGGACA	243
	R	TGAGATGGACTCTCCCATCC	
*COL13A1*	F	CAAAGGGAGAAGCAGGTGTC	175
	R	TCCTGGAGAGCCTCATTGAT	
